# Outcomes of Renal Transplantation in HIV-1 Associated Nephropathy

**DOI:** 10.1371/journal.pone.0129702

**Published:** 2015-06-10

**Authors:** Sana Waheed, Ahmad Sakr, Neha D. Chheda, Gregory M. Lucas, Michelle Estrella, Derek M. Fine, Mohamed G. Atta

**Affiliations:** 1 Medicine, University of Wisconsin School of Medicine, Madison, Wisconsin, United States of America; 2 Ain Shams Faculty of Medicine, Cairo, Egypt; 3 Medicine, Johns Hopkins School of Medicine, Baltimore, Maryland, United States of America; University of North Texas Health Science Center, UNITED STATES

## Abstract

**Introduction:**

Several studies have demonstrated that renal transplantation in HIV positive patients is both safe and effective. However, none of these studies have specifically examined outcomes in patients with HIV-associated nephropathy (HIVAN).

**Methods:**

Medical records of all HIV-infected patients who underwent kidney transplantation at Johns Hopkins Hospital between September 2006 and January 2014 were reviewed. Data was collected to examine baseline characteristics and outcomes of transplant recipients with HIVAN defined pathologically as collapsing focal segmental glomerulosclerosis (FSGS) with tubulo-interstitial disease.

**Results and Discussion:**

During the study period, a total of 16 patients with HIV infection underwent renal transplantation. Of those, 11 patients were identified to have biopsy-proven HIVAN as the primary cause of their end stage renal disease (ESRD) and were included in this study. They were predominantly African American males with a mean age of 47.6 years. Seven (64%) patients developed delayed graft function (DGF), and 6 (54%) patients required post-operative dialysis within one week of transplant. Graft survival rates at 1 and 3 years were 100% and 81%, respectively. Acute rejection rates at 1 and 3 years were 18% and 27%, respectively. During a mean follow up of 3.4 years, one patient died.

**Conclusions:**

Acute rejection rates in HIVAN patients in this study are higher than reported in the general ESRD population, which is similar to findings from prior studies of patients with HIV infection and ESRD of various causes. The high rejection rates appear to have no impact on short or intermediate term graft survival.

## Introduction

Kidney disease is a major cause of morbidity and mortality in patients with HIV-1 infection [1]. With progressive renal dysfunction, these patients often require renal replacement therapy. Although long-term survival on hemodialysis is possible in patients with HIV, their overall prognosis is worse compared to the general ESRD population [2,3].

Transplantation was previously considered to be high risk in these patients because of concerns regarding use of immunosuppression in the setting of dysregulated immune system. However, there has been increasing evidence that renal transplantation is both safe and effective in HIV-infected patients [4–10]. Interestingly, despite the overall suppressed immune system of these patients, rates of rejection are reported to be higher at 1 and 3 years in this population compared to kidney transplant recipients without HIV infection [11].

However, outcome data in all these studies were generated based on evaluation of kidney transplant recipients with various causes of ESR. None of the studies specifically examined transplant recipients with HIV-associated nephropathy (HIVAN) which is the most aggressive form of HIV-related kidney disease. In this study, we examined the impact of HIVAN on graft and patient survival in HIV-1 infected patients who underwent kidney transplantation at a large tertiary care center from 2006 to 2014.

## Material and Methods

### Patient population

The electronic medical records of all patients who underwent kidney transplantation at Johns Hopkins Hospital between September 2006 and January 2014 were reviewed. Patients who were older than 18 years of age, had evidence of HIV-1 infection and had biopsy-proven HIVAN as the etiology of their ESRD were included. All patients met the inclusion criteria for guidelines regarding renal transplantation and had stable maintenance on highly active antiretroviral therapy (HAART), undetectable plasma HIV-1 RNA levels, absolute CD4 counts of 200 cells/mm^3^ or more and absence of AIDS defining illness [12]. A total of 11 patients were identified who met the inclusion criteria.

### Ethics Statement

None of the transplant donors were from a vulnerable population and all donors or next of kin had provided written informed consent that was freely given prior use of organ.

### Data collection

Electronic transplant records, pathology reports for all biopsies, clinical follow-up data and laboratory values were abstracted. Patients were classified as having diabetes mellitus, hypertension and hypercholesterolemia based on diagnosis documented on medical records at the time of transplant evaluation. Co-existent hepatitis C viral infection was diagnosed if patients had detectable hepatitis C viral RNA at the time of diagnosis.

Serum creatinine was recorded on a monthly basis for the first year post-transplant and every 3 months thereafter as per clinical protocol. The etiology of patients’ cause of ESRD was determined by their native kidney biopsy pathology report. Patients with histologic evidence of collapsing focal segmental glomerulosclerosis and tubulointerstitial disease were classified as having HIVAN. Our selection excluded other histological reported cases of HIVAN without collapsing FSGS. HIV status was monitored by measurement of plasma HIV-1 viral copies and CD4 counts.

### Infection prophylaxis

Recorded infection prophylaxis medications for 180 days post-transplantation were similar to those used in kidney transplant recipients without HIV infection and included ganciclovir or valgancyclovir for cytomegalovirus, trimethoprim/ sulfamethoxazole or dapsone for *Pneumocystis carinii*, and fluconazole for *Candida albicans*.

### Definitions

Donors were classified as CDC high risk if they were incarcerated, used intravenous drugs, engaged in high-risk sexual behaviors, had hemophilia requiring human derived factor replacement or were exposed to HIV in the last twelve months [13]. The functional definition of delayed graft function (DGF) was used instead of the traditional dialysis based definition as it has been shown to be a more objective and superior metric for allograft outcomes. As such, DGF was defined as failure of serum creatinine levels to decrease by at least 10% daily on three consecutive days during the first post-operative week after transplantation [14]. Post-operative dialysis was defined as dialysis requirement within the first week after transplantation which is the traditional definition of DGF used in most of the other studies. Patients were classified as having acute cellular rejection if they had findings compatible with cellular rejection on a renal biopsy and were treated with thymoglobulin or high-dose steroids. Similarly, a diagnosis of antibody-mediated rejection was made based on renal biopsy findings and treatment with steroids, plasmapheresis or rituximab. If the patient became dialysis-dependent for more than 3 months, they were categorized as having developed graft failure.

### Statistical Methods

Stata statistical software version 12 (Stata Corp, College Station, TX) was used for the statistical analysis. Continuous variables were reported as mean ± standard deviation (SD). Continuous and categorical variables were compared using t-test and the Fisher’s exact test, respectively. A *P*-value <0.05 was considered statistically significant. We used Kaplan-Meier survival analysis to determine the time to ESRD, rejection, or death in patients with HIVAN. Patients were censored at the end of study, loss of follow up, death, or requirement of long term chronic dialysis. The study was approved by the Johns Hopkins Institutional Review Board.

## Results

### Baseline Characteristics

Of all the kidney transplants performed at Johns Hopkins Hospital between September 2006 and January 2014, 16 patients had HIV infection. Of those 16 patients, 2 patients had contracted HIV in the post-transplant period and 3 patients had an alternate cause for their ESRD. A total of 11 recipients had biopsy-proven HIVAN as the underlying cause of their ESRD and they were the subjects of this study. The characteristics of these 11 recipients are shown in Table 1. Most of the patients were African American males (91% and 82%, respectively). At the time of transplantation, mean baseline CD4 count was 606 cells/mm^3^ and the viral load was undetectable in all patients. Four patients (36%) were co-infected with hepatitis C virus.

**Table 1:** Characteristics of patients with HIVAN.

Characteristics	Numbers
Patients	11
Demographics	
Age [Mean(SD)]	47.6 (15.4)
Male [Number (%)]	9 (82)
Body Mass Index kg/m^2^, [Mean (SD)]	28.1 (5.8)
African American [Number (%)]	10 (91)
Diabetes Mellitus [Number (%)]	0 (0)
Hypertension [Number (%)]	9 (82)
Current Smoker [Number (%)]	1 (10)
Hyperlipidemia [Number (%)]	5 (45)
Hepatitis C infection [Number (%)]	4 (36.4)
ApoL-1 homozygosity[Number (%)]	3/3 (100)
Donor Related Variables	
Deceased Donor [Number (%)]	9 (82)
CDC High Risk Donors [Number (%)]	7 (63)
Cold Ischemia Time, hours [Mean (SD)]	22.6 (9.9)
Transplant Related Variables	
CD4 count [Mean (SD) cells/mm^3^]	606(243.6)
Delayed Graft Function [Number (%)]	7 (64)
Postoperative Dialysis [Number (%)]	6 (54)
Rejection [Number (%)]	4 (36)
CMV viremia [Number (%)]	0 (0)
Immunosuppression	
Induction with anti-thymocyte globulin [Number (%)]	10 (91)
HIV Treatment	
Non-Nucleoside Reverse Transcriptase Inhibitors [Number (%)]	8 (72.7)
Nucleoside Reverse Transcriptase Inhibitors [Number (%)]	10 (91)
Protease Inhibitors [Number (%)]	3 (27.3)
Integrase Inhibitors [Number (%)]	4 (36.4)

### Transplant Variables and Immunosuppression

As shown in Table 1, the majority of patients received kidneys from deceased donors. Only two patients had living donors, one of them was ABO-incompatible. Moreover, seven of the nine deceased donors were CDC-high risk. Thymoglobulin was the main induction agent used except for the use of daclizumab in one patient who underwent an ABO-incompatible transplant. The maintenance immunosuppressive regimen for all patients consisted of tacrolimus, mycophenolate mofetil or mycophenolic acid, and prednisone. The dose of tacrolimus was adjusted to a trough level of 8–12 ng/ml in the first 6 months after transplant. Seven (64%) patients developed DGF and six (54%) patients required post-operative dialysis. Table 2 describes the clinical attributes for the recipients and donors.

**Table 2:** Clinical Attributes of all HIVAN renal transplant recipients and their donors.

Patient No	Age at transplant (yrs), Sex, Race, BMI (kg/m²)	Pre-transplant CD4 count (per mm³), HIV RNA level (copies/ml)	HAART Regimen	HCV Co-infection	Donor age (yr), Type, CDCHR (Yes or No), CIT (hrs), Tcr (mg/dl)	Induction & Maintenance	DGF, Post-op dialysis	Details of rejection	Graft Loss and its timing	*Death and its cause*
***1***	53, M, AA, 25	878, <50	NNRTI, NRTI	Yes	?, DC, Yes, 6, 0.9	ATG. CNI, MMF, steroid	Yes, Yes	15 months post-transplant—ACR BANFF 2B; treated with ATG; suspected non-adherence.	Yes 1.5yrs post-transplant	No
***2***	52, F, AA, 35	949, <50	NNRTI, NRTI, INSTI	No	48, DC, Yes, 27, 0.7	ATG. CNI, MMF, steroid	No, No	No rejection	No	No
***3***	37, M, AA, 35	534, <50	NRTI, NNRTI, PI	No	24, DC, Yes, 9, 1.3	ATG. CNI, MMF, steroid	No, No	1 month post-transplant- ACR BANFF 2A; treated with ATG. 3 years post-transplant-AMR and ACR BANFF 2A—treated with plasmapharesis, rituximab, velcade, steroids. 4yr later—AMR	Yes 4.75 yrs post-transplant	No
***4***	50, M, AA, 21	502, <50	NNRTI, NRTI	Yes	20, DC, Yes, 21, 1	ATG. CNI, MMF, steroid	No, No	No rejection	No	Yes from toxic ingestion
***5***	40, F, AA, 24	1002, <50	NNRTI, NRTI	No	?, DC, No, 30, 0.9	ATG. CNI, MMF, steroid	Yes, No	No rejection	No	No
***6***	41, M, AA, 33	294, <50	NRTI, INSTI	No	?, DC, Yes, 27, 1.2	ATG. CNI, MMF, steroid	Yes, Yes	1 week post-transplant—ACR BANFF 2A; treated with ATG		No
***7***	38, M, AA, 37	688, <50	NNRTI, NRTI	No	21, DC, No, 25, 0.7	ATG. CNI, MMF, steroid	Yes, Yes	No rejection	No	No
***8***	49, M, AA, 27	575, <50	NRTI, PI, INSTI	No	52, DC, No, 17, 0.8	ATG. CNI, MMF, steroid	Yes, Yes	1 month post-transplant—ACR BANFF 2B and chronic AMR; treated with IVIG, plasmapharesis and ATG. 2 months later—AMR and BANFF 2A. 1 year later—acute and chronic AMR	No	No
***9***	61, M, AA, 23	493, <50	NNRTI, NRTI	Yes	21, DC, Yes, 24, 0.6	ATG. CNI, MMF, steroid	Yes, Yes	No rejection	No	Yes from septic shock
***10***	46, M, AA, 27	418, <50	NNRTI, NRTI, INSTI	Yes	48, DC, No, 40, 2.7	ATG. CNI, MMF, steroid	Yes, Yes	No rejection	No	No
***11***	57, M, C, 22	331, <50	NNRTI, NRTI, PI	No	LR	Daclizumab. CNI, MMF, steroid	No, No	No rejection	No	Yes

M, Male; F, Female; AA, African American; C, Caucasian; BMI, Body mass index,? not available; NNRTI, non-nucleoside reverse transcriptase inhibitor; NRTI, nucleoside reverse transcriptase inihibitor; PI, protease inhibitor; INSTI, integrase strand transfer inhibitor; CDCHR, CDC high risk; CIT, Cold ischemia time; Tcr, terminal creatinine; DC, deceased; LR, living related; ATG, anti-thymocyte globulin; CNI, calcineurin inhibitor; MMF, mycophenolate mofetil.

### Long-term outcomes

The mean follow up duration was 3.4 years (SD 2.1 years). As shown in Table 3, mean serum creatinine was 1.48mg/dl and 2.01mg/dl at 12 and 24 months of follow-up. Graft survival at 1 and 3 years was 100% and 81%. Four patients developed acute cellular or antibody-mediated rejection during the follow-up period. 37% of patients developed major complications as described below. Two patients progressed to graft failure requiring long-term dialysis. One patient developed squamous cell cancer of the anal canal approximately 4 years after transplantation. One patient died during the follow-up period from sepsis leading to multi-organ failure.

**Table 3:** Long-term outcomes of renal transplantation in patients with HIVAN.

Characteristics	Value
Mean serum creatinine at 12 months post-transplant, mg/dl (SD)	1.48 (0.5)
Mean serum creatinine at 24 months post-transplant, mg/dl (SD)	2.0(1.2)
One-year graft survival (%)	100%
Three-year graft survival (%)	81%
Long term dialysis [Number (%)]	2(18)
Graft failure [Number (%)]	2(18)
One-year rejection rate (%) [95% CI]	18 (4–53)
Three-year rejection rate (%) [95% CI]	27 (39–93)

ABO-incompatible transplant has been reported to be successful in HIV patients [15]. One patient included in our cohort underwent an ABO-incompatible transplant. This was a Caucasian patient who had developed ESRD due to biopsy- proven HIVAN. He underwent his first renal transplantation in 2000 from a living unrelated donor who was also of a Caucasian race. He developed graft failure secondary to recurrent biopsy-proven HIVAN and chronic allograft nephropathy. His HIV viral load was undetectable and CD4 count was >200 for many years preceding the diagnosis of recurrent HIVAN. Our first encounter with this patient was in late 2008 when he presented for an evaluation for an ABO-incompatible transplant. In early 2009, he received an HLA- and ABO-incompatible transplant from his brother. Brother’s blood group was AB and patient’s blood group was O with an anti-AB titer of 256. After six pre-transplant plasmapheresis treatments, he received one dose of rituximab followed by daclizumab for induction. Post-transplant, he received another four sessions of plasmapheresis and was started on prednisone, mycophenolate mofetil and tacrolimus for maintenance immunosuppression. He was continued on a PI based HAART regimen. Tacrolimus was subsequently stopped due to findings of tacrolimus toxicity on a renal biopsy; however, he continued to have stable graft function with a serum creatinine of 1.3mg/dl. This patient had no episodes of acute cellular rejection unlike the other HIV patient with an ABO-incompatible renal transplant reported by Campara et al. who had two episodes of acute cellular rejection with creatinine stabilizing at 2.0mg/dl [15]. Unfortunately, this patient developed recurrent anal cancer and passed away in 2013. His serum creatinine remained stable at 1.17 mg/dl on the day of death.

## Discussion

Based on this retrospective analysis of a cohort of biopsy-proven HIVAN patients with renal transplantation, we report that despite higher rates of acute rejection and DGF, intermediate term renal allograft survival in HIVAN patients is possible. The number of biopsy-proven HIVAN patients examined in our study, although small, is higher than those in previous studies of HIV infected renal transplant recipients which included patients with ESRD from various causes and the diagnosis of HIVAN was presumed rather than biopsy confirmed [8,11].

**Fig 1:**
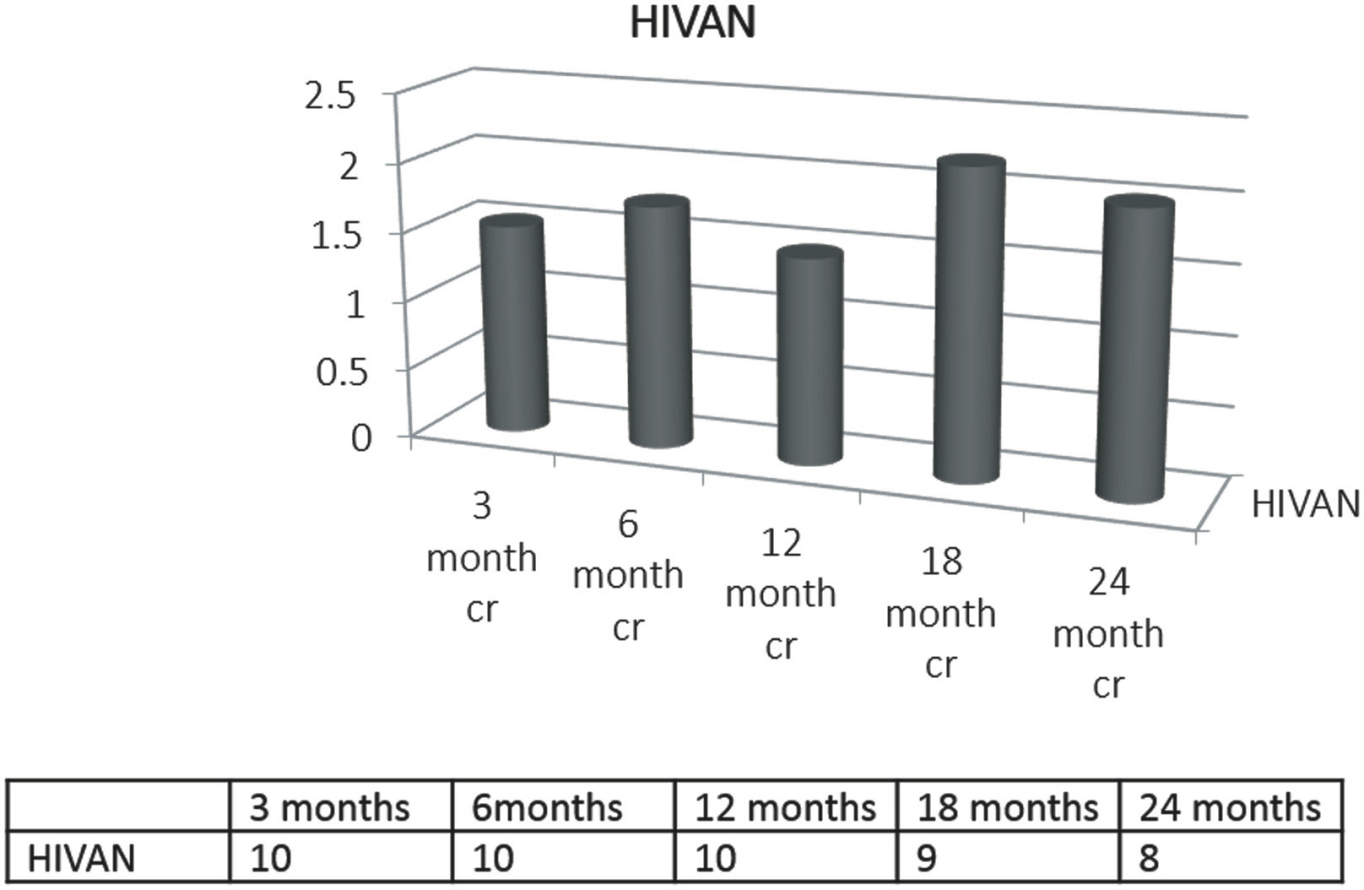
Mean Serum Creatinine (mg/dl) at 3, 6, 12, 18, and 24 months post-transplant in patients with HIVAN.

In general, DGF rates are reported to be higher in kidney transplant patients who are HIV positive compared to HIV-1 negative patients [16]. In our study, DGF rate for HIVAN patients was 64% which is similar to what has been demonstrated in a recent Spanish study of HIV-infected renal transplant recipients, where DGF rate was 60% [16]. This high rate of DGF could be secondary to the underlying inflammatory state in patients with HIVAN but future studies are needed to elucidate this observation further. Obesity has recently been demonstrated as a risk factor for DGF; however, it was not a prevalent problem in our cohort [17]. Despite a high rate of DGF, the mean serum creatinine at 24 months of follow-up was stable around 2 mg/dl (Fig 1). These results corroborate what has been recently demonstrated in a study of 40 HIV-infected patients, in which the mean serum creatinine was 2.2mg/dl at 24 months of follow-up [8]. In our cohort, the 3-year graft survival was 81%, which is slightly higher than what was reported in prior studies of HIV patients with ESRD from various causes although this is not statistically significant. In the largest study by Stock et al. of 150 HIV-1 infected patients who underwent renal transplantation, graft and patient survival rates were 90.4% and 94.6% at 1 year and 73.7% and 88.2% at 3 years, respectively [11].

The first prospective study of HIV positive kidney transplant patients in the HAART era included 10 patients who did not receive any induction therapy. Although this study demonstrated similar 1-year patient and graft survival rates to patients without HIV infection, more than half of the HIV positive patients experienced acute rejection [6]. Among kidney allograft recipients in our cohort, the 1- and 3-year rejection rates were 18% and 27%. These rates are much higher than the general renal transplant population as reported in the Scientific Registry of Transplant Recipients [18]. Higher rejection rates in HIV-infected patients have been demonstrated in prior studies as well. Recent studies have shown that despite undetectable serum HIV RNA levels, HIV can be detected in 68% of renal allografts specifically in podocytes and tubular cells. This can possibly account for higher rates of rejection and decline in allograft function [19]. Another possible explanation for higher rejection rates is inadequate immunosuppression. Calcineurin inhibitors (CNIs) are administered less frequently in HIV-infected kidney transplant recipients because of drug-drug interaction with protease inhibitors (PIs), which potently inhibit the cytochrome p-450 3A4 system [7]. One of our patients was only on 0.125mg of tarcolimus every other day compared to a total daily dose of 4–6 mg, which is normally used in non-HIV patients. The pharmokinetic curves of CNIs in patients who are on PIs do not show the typical peaks and troughs and rather have a flattening of the curves. Therefore, even though the measured trough levels of CNIs are therapeutic, the total exposure might be sub-therapeutic in these patients [7].

Antithymocyte globulin (ATG) was the induction agent used in all but one of our patients and there were no serious infections in the immediate post-operative period. Concerns regarding the use of ATG for induction have been raised as a T cell depleting agent in patients with already low CD4 counts might increase the risk of infections, but this increased risk has never been demonstrated in a prospective trial [20]. In a recent study of 516 HIV-infected patients, ATG use for induction was as associated with a 2.6-fold lower risk of acute rejection (RR, 0.39, P = 0.02) compared to patients receiving no antibody induction [21]. Mycophenolate mofetil was used as a maintenance immunosuppressive medication in all our patients which has known virostatic action against HIV by depletion of guanoside nucleosides that is required for viral replication [22]. Although sirolimus was not used in any of our patients, recent studies have shown an association between sirolimus use and low HIV RNA levels post-transplant [23]. With the use of maintenance immunosuppression, there was no evidence of HIV disease progression in our patients. Despite challenging drug interactions, HIV viral load remained well controlled. None of these patients had recurrence of HIVAN. Presumably most of these patients carried the ApoL1 risk variant even though it was confirmed only in 3 patients. Absence of recurrent HIVAN is not an unexpected finding since it is hypothesized that the risk caused by ApoL1 will be transmitted with the donor kidney and would not be based on the recipient’s genotype [24].

The major strength of our study is that the outcomes of patients with HIVAN were analyzed separately. We acknowledge the small sample size as the main limitation of our study although this is the first study that specifically explores graft and patient survival of kidney transplant in biopsy-proven HIVAN recipients. It is also important to note that our sample was restricted to only those defined pathologically as collapsing focal segmental glomerulosclerosis (FSGS) with tubulo-interstitial disease excluding other histological reported cases of HIVAN without collapsing FSGS, including the first cases of HIVAN reported in 1984, and in many other studies.

In conclusion, kidney transplant appears effective in patients with HIVAN despite elevated rates of DGF. Further studies are needed to evaluate the reason for higher rates of DGF which were also seen in our cohort. Moreover, the safety of ABO-incompatible kidney transplant in this population requires further evaluation.
